# Increased iNOS and Nitrosative Stress in Dopaminergic Neurons of MDMA-Exposed Rats

**DOI:** 10.3390/ijms20051242

**Published:** 2019-03-12

**Authors:** Stefania Schiavone, Margherita Neri, Angela Bruna Maffione, Paolo Frisoni, Maria Grazia Morgese, Luigia Trabace, Emanuela Turillazzi

**Affiliations:** 1 Department of Clinical and Experimental Medicine, University of Foggia, Via Napoli, 20, 71122 Foggia, Italy; angelabruna.maffione@unifg.it (A.B.M.); mariagrazia.morgese@unifg.it (M.G.M.); luigia.trabace@unifg.it (L.T.); 2 Department of Morphology, Surgery and Experimental Medicine, University of Ferrara, Via Fossato di Mortara, 70, 44100 Ferrara, Italy; margherita.neri@unife.it (M.N.); paolo.frisoni@unife.it (P.F.); 3 Section of Legal Medicine, Department of Surgical, Medical, Molecular and Critical Pathology, University of Pisa, Via Roma 55, 56126 Pisa, Italy; emanuela_turillazzi@inwind.it

**Keywords:** MDMA, oxidative stress, nitrosative stress, iNOS, NADPH oxidases

## Abstract

Several mechanisms underlying 3,4-Methylenedioxy-*N*-methylamphetamine (MDMA) neurotoxicity have been proposed, including neurochemical alterations and excitotoxicity mediated by reactive oxygen species (ROS), nitric oxide (NO), and reactive nitrogen species (RNS). However, ROS, NO, and RNS sources in the brain are not fully known. We aimed to investigate possible alterations in the expression of the ROS producer NOX enzymes (NOX2, NOX1, and NOX4), NO generators (iNOS, eNOS, and nNOS), markers of oxidative (8-hydroxy-2′-deoxyguanosine, 8OHdG), and nitrosative (3-nitrotyrosine, NT) stress, as well as the colocalization between cells positive for the dopamine transporter (DT1) and cells expressing the neuronal nuclei (NeuN) marker, in the frontal cortex of rats receiving saline or MDMA, sacrificed 6 h, 16 h, or 24 h after its administration. MDMA did not affect NOX2, NOX1, and NOX4 immunoreactivity, whereas iNOS expression was enhanced. The number of NT-positive cells was increased in MDMA-exposed animals, whereas no differences were detected in 8OHdG expression among experimental groups. MDMA and NT markers colocalized with DT1 positive cells. DT1 immunostaining was found in NeuN-positive stained cells. Virtually no colocalization was observed with microglia and astrocytes. Moreover, MDMA immunostaining was not found in NOX2-positive cells. Our results suggest that iNOS-derived nitrosative stress, but not NOX enzymes, may have a crucial role in the pathogenesis of MDMA-induced neurotoxicity, highlighting the specificity of different enzymatic systems in the development of neuropathological alterations induced by the abuse of this psychoactive compound.

## 1. Introduction

3,4-Methylenedioxy-*N*-methylamphetamine (MDMA), also known as ecstasy, is a synthetic entactogen of the phenethylamine family, used primarily as an illicit recreational drug, especially in the context of rave culture [1]. The use of MDMA is highly alarming and, therefore, there is an urgent need to deeply understand the consequences of MDMA consumption. Neurotoxicity is one of them and evaluations of MDMA effects on human psychobiology have, consequently, been the focus of several studies [2,3,4,5,6]. Since studies on humans have ethical barriers, investigation on MDMA neurotoxicity in animal models have been widely performed and are of extreme importance. Indeed, the neurotoxic and neuroinflammatory effects associated with the abuse of this psychoactive compound have been widely described in rodent studies, especially in terms of altered dopaminergic neurotransmission and long-term degeneration of dopaminergic nerves [7,8,9].

The dopamine transporter (DT1), a marker of dopaminergic neurons [10], has also been described as a major target for drugs of abuse [11]. In particular, a reduction of its density has been observed in MDMA recreational users [12]. Preclinical evidence, obtained by using mice lacking this transporter, also highlighted that it is an essential mediator required for methamphetamine-induced neurotoxicity [13]. Moreover, MDMA-exposed rats showed a dose-dependent decrease in the striatal binding of this transporter [14].

Recent evidence has highlighted that, besides its detrimental effects on the striatum and the pars compacta of the substantia nigra, MDMA also affects cortical regions, altering the firing pattern of dopamine neurons, probably via an increase of norepinephrine release, finally resulting in dopamine release alterations [15]. Importantly, dysfunctions of the frontal cortex have been described to be directly implicated in memory deficits observed in MDMA users [16]. Alterations in the expression of specific gene sets related to protein synthesis, transmembrane transport processes, and synaptic plasticity have also been reported in the frontal cortex of rats following MDMA administration [17]. Moreover, MDMA binge consumption in rodents induced alterations of serotonergic and dopaminergic neurotransmission in this brain region [14].

Several pathogenetic mechanisms underlying MDMA-neurotoxicity have been proposed, including excitotoxicity, mediated by the increased production of reactive oxygen species (ROS), nitric oxide (NO), and reactive nitrogen species (RNS) [18,19,20,21], leading ultimately to excessive glutamate release, activation of specific glutamate receptors, elevation of intracellular calcium levels, and activation of apoptotic processes [22]. However, despite the increasing amount of evidence in this sense, the sources of ROS, NO, and RNS resulting from MDMA exposure are not fully known. One of the major sources of ROS in the central nervous system (CNS) is represented by the family of the nicotinamide adenine dinucleotide phosphate (NADPH) oxidase NOX enzymes, which have been described as crucial contributors of several physiological functions in the brain, as well as key players in the pathogenesis of different CNS diseases, going from neurodegenerative to psychiatric pathologies [23,24]. In the context of psychoactive substance abuse, the NOX2 enzyme has been implicated in human cocaine-induced neurotoxicity [25], also associated with the excited delirium syndrome [26], as well as in the development of neuropathological alterations induced by ketamine administration in mice [27,28]. With respect to NO production in the CNS, the three isoforms of the NO synthase (NOS) enzyme (i.e., neuronal (nNOS), inducible (iNOS), and endothelial (eNOS)) have been widely considered the most important NO source [29] in both physiological and pathological CNS conditions [30,31,32,33]. 

Here, by using an immunohistochemical approach, we investigated possible alterations in the expression of NOX2, NOX1, and NOX4 enzymes as ROS sources, NO producers iNOS, eNOS, and nNOS, as well as 8-hydroxy-2′-deoxyguanosine (8OHdG, a marker of oxidative stress) and 3-nitrotyrosine (NT, a marker of nitrosative stress) in the frontal cortex of rats receiving saline or MDMA administration and sacrificed at three different time points, namely, 6 h, 16 h, and 24 h from the injection of this psychoactive compound. Finally, we also evaluated the possible involvement of dopaminergic neurons, microglia, and astrocytes in MDMA-induced immunohistochemical alterations.

## 2. Results

### 2.1. MDMA Administration Did Not Affect NADPH Oxidase Immunoreactivity

**Figure 1:**
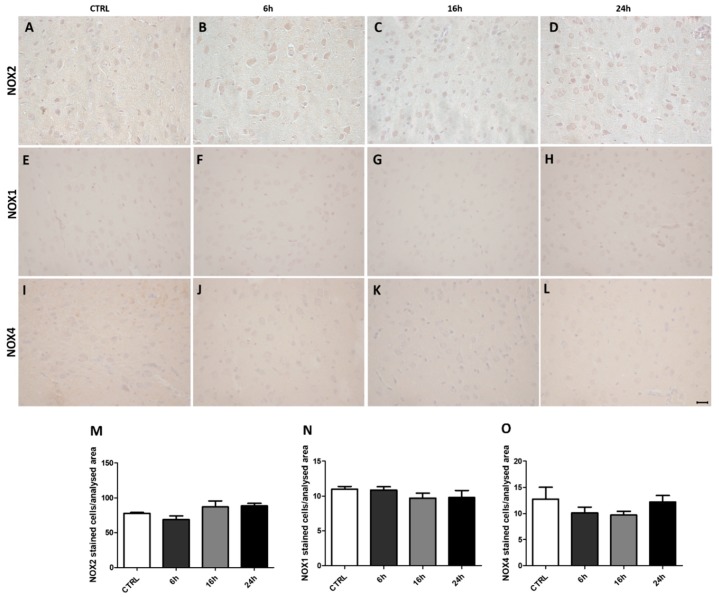
Nicotinamide adenine dinucleotide phosphate (NADPH) oxidase immunoreactivity is not affected by 3,4-Methylenedioxy-*N*-methylamphetamine (MDMA) administration. (**A**–**D**) Representative images (light microscopy, 40×) of NOX2 immunoreactivity in the frontal cortex of rats receiving (**A**) saline (controls = CTRL) and of rats receiving MDMA and sacrificed after (**B**) 6 h, (**C**) 16 h, and (**D**) 24 h from its administration. (**E**–**H**) Representative images (light microscopy, 40×) of NOX1 immunoreactivity in the frontal cortex of rats receiving (**E**) saline (CTRL) and of rats receiving MDMA and sacrificed after (**F**) 6 h, (**G**) 16 h, and (**H**) 24 h from its administration. (**I**–**L**) Representative images (light microscopy, 40×) of NOX4 immunoreactivity in the frontal cortex of rats receiving (**I**) saline (CTRL) and of rats receiving MDMA and sacrificed after (**J**) 6 h, (**K**) 16 h, and (**L**) 24 h from its administration. Scale bar for images in panels (**A**–**L**) = 50 μm. (**M**–**O**) Quantification of (**M**) NOX2, (**N**) NOX1, and (**O**) NOX4 positive-stained cells/area analyzed in controls (CTRL) and MDMA-exposed rats, sacrificed after 6 h, 16 h, and 24 h from its administration. One-way ANOVA followed by Tukey’s post-hoc test. For NOX2: F = 2.281, *p* ˃ 0.05; for NOX1: F = 0.9608, *p* ˃ 0.05; for NOX4: F = 1.333, *p* ˃ 0.05.

In order to assess if MDMA administration could induce modifications in the expression of the NADPH oxidase NOX enzymes, we analyzed NOX2, NOX1, and NOX4 immunoreactivities in the frontal cortex of control (CTRL) and rats sacrificed after 6 h, 16 h, and 24 h from MDMA administration. Immunohistochemical results showed no significant differences in NOX2 expression between CTRL and rats sacrificed after the three different time points from MDMA administration, as well as within the MDMA-exposed group (Figure 1A–D). This was confirmed by the correspondent quantifications of NOX2-positive stained cells (Figure 1M, one-way ANOVA followed by Tukey’s post-hoc test, F = 2.281, *p* ˃ 0.05). The same was observed for NOX1 (Figure 1E–H) and NOX4 (Figure 1I–L) immunoreactivities and pertaining quantifications (for NOX1 (Figure 1N), one-way ANOVA followed by Tukey’s post-hoc test, F = 0.9608, *p* ˃ 0.05; for NOX4 (Figure 1O), one-way ANOVA followed by Tukey’s post-hoc test, F = 1.333, *p* ˃ 0.05).

### 2.2. MDMA Administration Induced an Increase in iNOS Immunoreactivity

**Figure 2:**
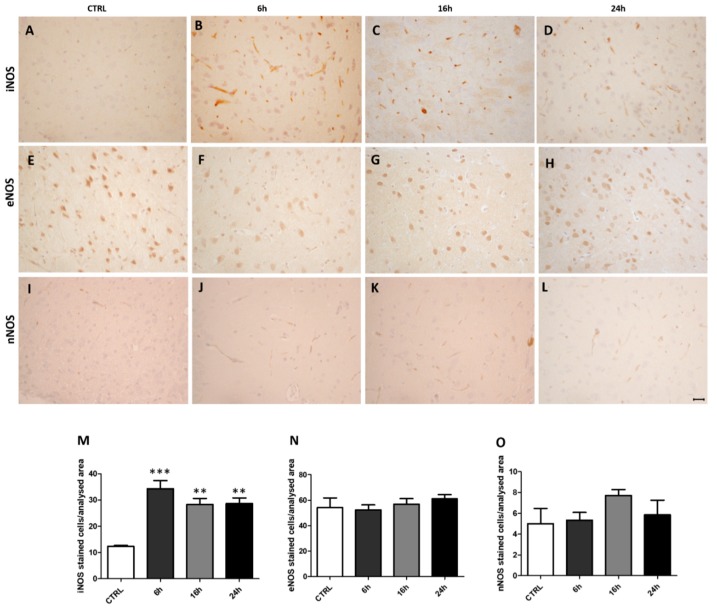
Inducible nitric oxide synthase (iNOS) immunoreactivity is increased following MDMA administration. (**A**–**D**) Representative images (light microscopy, 40×) of iNOS immunoreactivity in the frontal cortex of rats receiving (**A**) saline (CTRL) and of rats receiving MDMA and sacrificed after (**B**) 6 h, (**C**) 16 h, and (**D**) 24 h from its administration. (**E**–**H**) Representative images (light microscopy, 40×) of endothelial nitric oxide synthase (eNOS) immunoreactivity in the frontal cortex of rats receiving (**E**) saline (CTRL) and of rats receiving MDMA and sacrificed after (**F**) 6 h, (**G**) 16 h, and (**H**) 24 h from its administration. (**I**–**L**) Representative images (light microscopy, 40×) of neuronal nitric oxide synthase (nNOS) immunoreactivity in the frontal cortex of rats receiving (**I**) saline (CTRL) and of rats receiving MDMA and sacrificed after (**J**) 6 h, (**K**) 16 h, and (**L**) 24 h from its administration. Scale bar for images in panels (**A**–**L**) = 50 μm. (**M**–**O**) Quantification of (**M**) iNOS, (**N**) eNOS, and (**O**) nNOS positive-stained cells/area analyzed in controls (CTRL) and MDMA-exposed rats, sacrificed after 6 h, 16 h, and 24 h from its administration. One-way ANOVA followed by Tukey’s post-hoc test. For iNOS: F = 9.090, *** *p* < 0.001 6 h vs. CTRL, ** *p* < 0.01 16 h vs. CTRL and 24 h vs. CTRL, *p* ˃ 0.05 6 h vs. 16 h, 6 h vs. 24 h, and 16 h vs. 24 h; for eNOS: F = 0.7276, *p* ˃ 0.05; for nNOS: F = 1.290, *p* ˃ 0.05.

In order to evaluate possible alterations of NOS induced by MDMA administration, we compared iNOS, eNOS, and nNOS expressions in the frontal cortex by immunohistochemistry between saline- and MDMA-exposed rats sacrificed after 6 h, 16 h, and 24 h. Inducible NOS immunoreactivity was significantly increased in MDMA-exposed animals with respect to CTRL, while no significant changes in iNOS expression was detected within the three MDMA-exposed groups (Figure 2A–D,M, one-way ANOVA followed by Tukey’s post-hoc test, F = 9.090, 6 h vs. CTRL *p* < 0.001, 16 h vs. CTRL *p* < 0.01, 24 h vs. CTRL *p* < 0.01, 6 h vs. 16 h *p* ˃ 0.05, 6 h vs. 24 h *p* ˃ 0.05, 16 h vs. 24 h *p* ˃ 0.05). No statistical differences were observed for eNOS expression both between and within the four experimental groups (Figure 2E–H,N, one-way ANOVA followed by Tukey’s post-hoc test, F = 0.7276, *p* ˃ 0.05). The same results were found for nNOS immunoreactivity and correspondent quantification (Figure 2I–L,O, one-way ANOVA followed by Tukey’s post-hoc test, F = 1.290, *p* ˃ 0.05).

### 2.3. MDMA Administration Induced an Increase in NT Immunoreactivity

**Figure 3:**
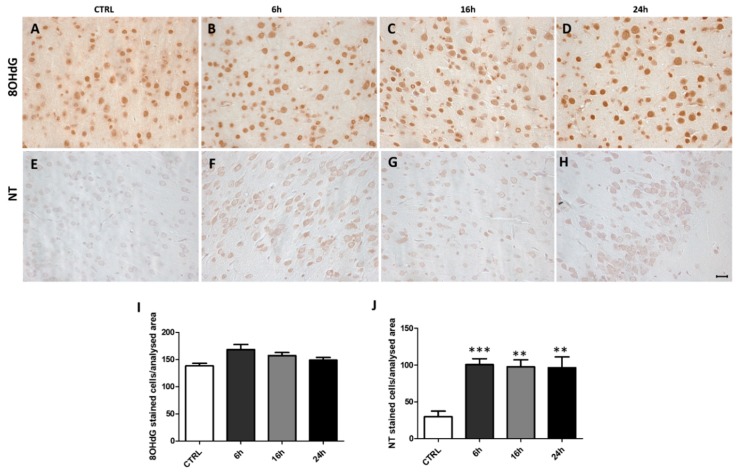
3-nitrotyrosine (NT) immunoreactivity is increased following MDMA administration. (**A**–**D**) Representative images (light microscopy, 40×) of 8-hydroxy-2′-deoxyguanosine (8OHdG) immunoreactivity in the frontal cortex of rats receiving (**A**) saline (CTRL) and of rats receiving MDMA and sacrificed after (**B**) 6 h, (**C**) 16 h, and (**D**) 24 h from its administration. (**E**–**H**) Representative images (light microscopy, 40×) of NT immunoreactivity in the frontal cortex of rats receiving (**E**) saline (CTRL) and of rats receiving MDMA and sacrificed after (**F**) 6 h, (**G**) 16 h, and (**H**) 24 h from its administration. Scale bar for images in panels (**A**–**H**) = 50 μm. (**I**–**J**) Quantification of (**I**) 8OHdG and (**J**) NT positive-stained cells/area analyzed in controls (CTRL) and MDMA-exposed rats, sacrificed after 6 h, 16 h, and 24 h from its administration. One-way ANOVA followed by Tukey’s post-hoc test. For 8OHdG: F = 3.141, *p* ˃ 0.05; for NT: F = 9.471, *** *p* < 0.001 6 h vs. CTRL, ** *p* < 0.01 16 h vs. CTRL and 24 h vs. CTRL, *p* ˃ 0.05 6 h vs. 16 h, 6 h vs. 24 h, and 16 h vs. 24 h.

To assess possible increase of biomarkers of nitrosative or oxidative stress, following MDMA administration, we performed immunohistochemical analysis for NT and 8OHdG, respectively, in the frontal cortex. While no differences were observed for 8OHdG immunoreactivity among the four experimental groups and within MDMA-exposed animals (Figure 3A–D,I, one-way ANOVA followed by Tukey’s post-hoc test, F = 3.141, *p* ˃ 0.05), a significant elevation in NT expression was identified between CTRL and MDMA-exposed rats (Figure 3E–H,J, one-way ANOVA followed by Tukey’s post-hoc test, F = 9.471, 6 h vs. CTRL *p* < 0.001, 16 h vs. CTRL *p* < 0.01, 24 h vs. CTRL *p* < 0.01, 6 h vs. 16 h *p* ˃ 0.05, 6 h vs. 24 h *p* ˃ 0.05, 16 h vs. 24 h *p* ˃ 0.05).

### 2.4. MDMA and NT Were Localized in Dopaminergic Neurons

**Figure 4:**
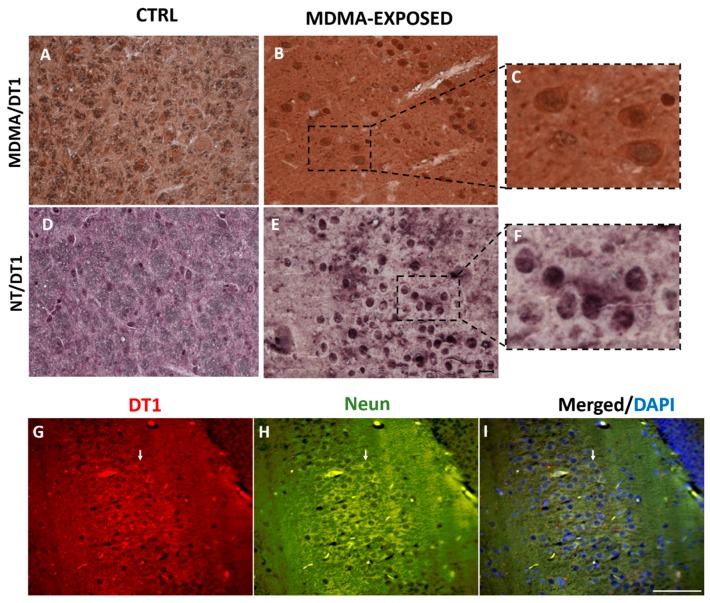
MDMA and NT colocalized with dopamine transporter (DT1). (**A**,**B**) Representative images (light microscopy, 40×) of MDMA/DT1 double immunostaining in the frontal cortex of rats receiving (**A**) saline (CTRL) and of rats receiving (**B**) MDMA (MDMA-exposed, sacrificed after 6 h). (**C**) Blown-up image of the dotted area highlighted in (**B**). (**D**,**E**) Representative images (light microscopy, 40×) of NT/DT1 double immunostaining in the frontal cortex of rats receiving (**D**) saline (CTRL) and in rats receiving (**E**) MDMA (MDMA-exposed, sacrificed after 6 h). (**F**) Blown-up image of the dotted area highlighted in (**E**). Scale bar for images in (**A**,**B**,**D**,**E**) = 50 μm. (**G**–**I**) Representative images (light microscopy, 40×) of DT1 (**G**-red staining)/NeuN (**H**-green staining) immunofluorescence and merged images with 4′, 6-diamidino-2-phenylindole (DAPI) (**I**-blue staining) in the frontal cortex. Scale bar for images in (**G**–**I**) = 100 μm. Arrows indicate a representative DT1/NeuN double-stained cell.

To determine a possible colocalization of MDMA and NT with dopaminergic neurons, double immunohistochemistry for MDMA or NT and DT1 was performed. Results showed that MDMA and DT1 immunoreactivities colocalized in the cortex of rats administered with this substance (Figure 4A–C and Figure S1A,B). Furthermore, NT-immunoreactive cells were also found to be double-stained with DT1 (Figure 4D–F and Figure S1C,D). DT1 immunofluorescence was detected in cells also expressing the neuronal nuclei (NeuN) marker (Figure 4G–I). 

### 2.5. MDMA and NT Were Not Localized in Glia Cells

**Figure 5:**
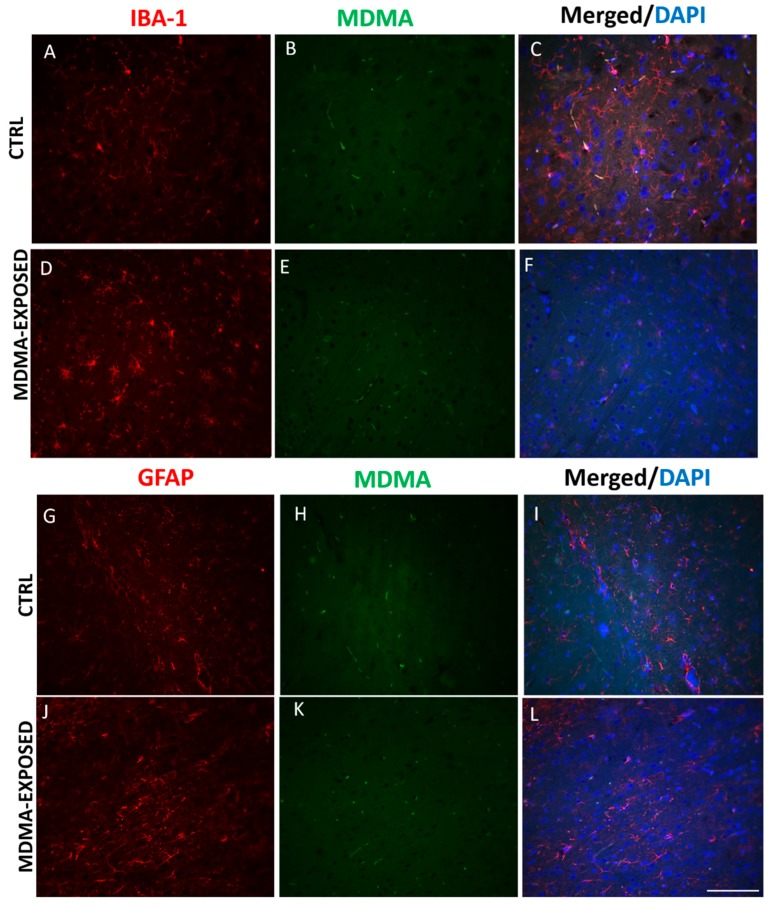
MDMA immunofluorescence is not present in microglia and astrocytes. (**A**–**F**) Representative immunofluorescence images (light microscopy, 40×) of ionized calcium-binding adapter molecule 1 (IBA-1) (red staining)/MDMA (green staining) and merged images with DAPI (blue staining) in the frontal cortex of (**A**–**C**) saline-exposed (CTRL) and (**D**–**F**) MDMA-exposed (sacrificed after 6 h from its administration) rats. (**G**–**L**) Representative immunofluorescence images (light microscopy, 40×) of glial fibrillary acidic protein (GFAP) (red staining)/MDMA (green staining) and merged images with DAPI (blue staining) in the frontal cortex of (**G**–**I**) saline-exposed (CTRL) and (**J**–**L**) MDMA-exposed (sacrificed after 6 h from its administration) rats. Scale bar for images in panels (**A**–**L**) = 100 μm.

To assess if MDMA and NT were also present in other CNS cellular subtypes, we performed double immunofluorescence experiments for MDMA or NT and ionized calcium-binding adapter molecule 1 (IBA-1), a marker of microglia [34] and for MDMA or NT and glial fibrillary acidic protein (GFAP), a marker of astrocytes [35]. Results showed that IBA-1 and GFAP immunofluorescence did not co-stain with MDMA (Figure 5A–L and Figure S2A–L).

**Figure 6:**
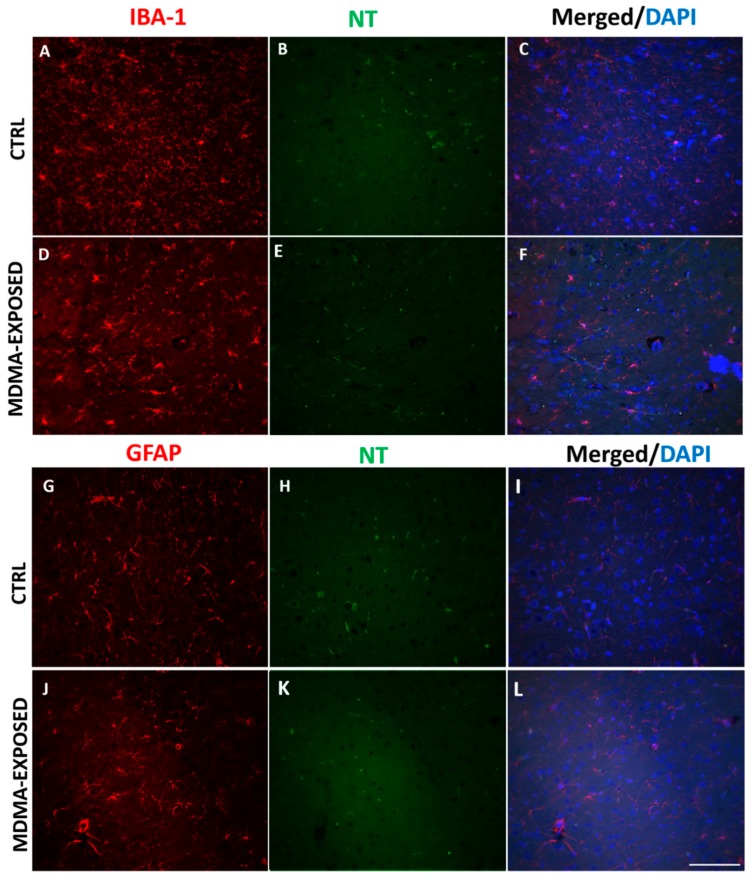
NT immunofluorescence is not present in microglia and astrocytes. (**A**–**F**) Representative immunofluorescence images (light microscopy, 40×) of IBA-1 (red staining)/NT (green staining) and merged images with DAPI (blue staining) in the frontal cortex of (**A**–**C**) saline-exposed (CTRL) and (**D**–**F**) MDMA-exposed (sacrificed after 6 h from its administration) rats. (**G**–**L**) Representative immunofluorescence images (light microscopy, 40×) of GFAP (red staining)/NT (green staining) and merged images with DAPI (blue staining) in the frontal cortex of (**G**–**I**) saline-exposed (CTRL) and (**J**–**L**) MDMA-exposed (sacrificed after 6 h from its administration) rats. Scale bar for images in panels (**A**–**L**) = 100 μm.

No colocalization was detected between IBA-1 and NT immunofluorescence as well as between GFAP and NT staining (Figure 6A–L and Figure S3A–L). 

**Figure 7:**
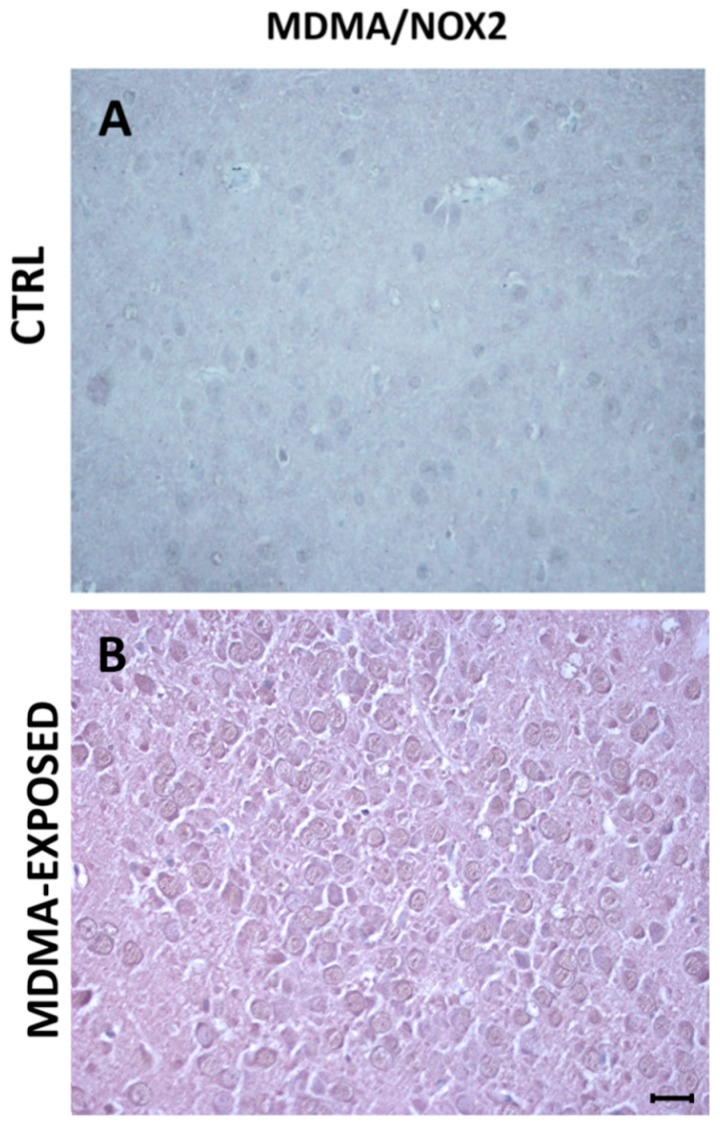
MDMA staining does not colocalize with NOX2 immunoreactivity. (**A**,**B**) Representative images (light microscopy, 40×) of MDMA/NOX2 double immunostaining in the frontal cortex of rats receiving (**A**) saline (CTRL) and of rats receiving (**B**) MDMA (sacrificed after 6 h from its administration). Scale bar for images in panels (**A**,**B**) = 50 μm.

Virtually, no colocalization was observed between MDMA immunoreactive and NOX2-positive stained cells (Figure 7A,B and Figure S4A,B).

## 3. Discussion

In this study, we investigated if MDMA may induce oxidative and/or nitrosative stress in the frontal cortex of rats. We found that MDMA did not affect NOX enzyme immunoreactivity in this brain region, whereas iNOS expression was enhanced along with the number of NT-positive cells. MDMA and NT markers were detected in DT1-stained cells but not in microglia and astrocytes.

The link between MDMA administration and a possible increase of oxidative stress in other organs, such as liver and heart, especially myocardium, has been previously investigated [36,37,38,39,40]. However, only a limited number of studies have evaluated the impact of MDMA on the brain redox state [41,42,43], in particular with respect to the identification of possible sources of free radical production. In regard to this point, we found that the immunoreactivity of NOX2, NOX1, and NOX4 as well as the expression of 8OHdG, a marker of ROS-induced oxidative damage to DNA [44] widely considered as a reliable biomarker for oxidative stress presence, were not increased in the frontal cortex following MDMA administration with respect to saline-exposed animals, at the three considered time points. These results are in line with our previous observations showing that NOX2 was not implicated in the alterations of neurotransmitter release and behavioral changes in response to amphetamine administration in mice [27]. Indeed, we previously found that amphetamine administration induced similar neurochemical and behavioral responses in wild-type and NOX2 knockout mice, while increased glutamate and dopamine release induced by subchronic administration of subanaesthetic doses of ketamine were abolished after NOX2 deficiency in mice [27]. These data suggest that this enzyme may not be directly implicated in the pathogenetic pathways underlying MDMA-induced neurotoxicity. They are also in line with previous works reporting an enhancement of free radical production by mitochondria [45,46,47] and an alteration of specific mitochondria-related biochemical parameters, following MDMA administration [48]. However, we cannot totally exclude a possible involvement of the NADPH oxidases in the pathogenesis of MDMA-induced neurotoxicity, also in the light of the role that the NADPH plays in the glutathione-dependent neutralization of MDMA-derived oxidized metabolites [49].

A novel finding of our study, if compared to previous lines of evidence showing a direct involvement of the nitrergic system in the onset and progression of MDMA-neurotoxicity [50,51,52], is the detection of iNOS immunoreactivity increase in the cortex of MDMA-exposed animals with respect to CTRL. Importantly, iNOS has been reported to act as a key modulator of neuronal death. Indeed, iNOS knockout mice injected with kainic acid showed a reduced number of TUNEL positive cells in the hippocampus [53]. In the same line, iNOS-derived NO was able to induce neuronal death following hypoxic-ischemic insults by interacting with NMDA receptors [54] or directly synergizing with hypoxia [55].

At least in our experimental conditions, there were no significant differences in nNOS expression between MDMA-exposed rats and CTRL. Accordingly, previous observations reported that the induction of psychomotor sensitization to MDMA was dependent upon NO and that repeated administration of MDMA resulted in psychomotor sensitization in both wild-type and nNOS knockout mice [56]. Moreover, an increased immunoreactivity for nNOS was observed in the striatum and nucleus accumbens, but not in the cortex, of mice following administration of repeated doses of MDMA [57]. An increased expression of nNOS has also been reported only following the administration of MDMA in association with other psychostimulant compounds, such as caffeine [58]. With respect to the lack of eNOS expression alterations in the brain of MDMA-exposed rats, our results are in line with a previously published paper reporting an involvement of eNOS in the pathological consequences of MDMA administration, combined with other molecules, such as phosphodiesterase 5 inhibitors, in peripheral body districts [59]. Besides the observed iNOS involvement, a possible implication of other sources of nitrosylating species, such as myeloperoxidase, could not be excluded. Indeed, this enzyme has been described to interact directly with iNOS, up-regulating its catalytic activity or consuming NO released by iNOS and therefore preventing the NO-induced inhibition attributed to the formation of the iNOS–nitrosyl complex [60]. Moreover, myeloperoxidase has been reported to be a crucial component of the MDMA-induced intracellular enzymatic cascade which leads to the activation of pro-apoptotic signals, finally resulting in toxic damage, cell dysfunction, and death [61].

We observed a colocalization between MDMA and DT1 staining, as well as between NT and DT1-positive cells. This is an important finding of our study, in line with previously published reports describing alterations of the dopaminergic system and neurotransmission induced by MDMA [62]. This is also particularly relevant with respect to the age of the animals we used (late adolescence/beginning of adulthood). Indeed, it has been demonstrated that adult rats exposed to MDMA during adolescence showed a significant reduction of dopamine cell bodies and terminals at adulthood, associated with a reduced density of TH-positive neurons, a decreased immunoreactivity dopamine transporter, a reduction of basal dopamine release, and a deficit in the processes of memory formation and recognition [63]. However, the possible implication of other neurotransmitters, such as serotonin (5-HT), should also be considered. In this regard, it has been reported that the 5-HT receptor subtypes differentially contribute to the behavioral effects of MDMA—the 5-HT2A and 5-HT1B/1D receptors playing a facilitatory role in mediating the stimulant effect of this molecule, the 5-HT2C being, instead, inhibitory [64]. Moreover, both rodent and clinical neuroimaging studies, conducted on frequent MDMA users, reported that this substance induces a massive 5-HT release, with consequent decrease of the 5-HT transporter binding in different brain regions [62,65,66,67]. These 5-HT-related alterations induced by MDMA administration should also be seen in the light of the physiological connections existing between the serotonergic and the dopaminergic systems. Indeed, a recent study, performed by combining cell-type-specific fiber photometry of Ca^2+^ signals and intravenous drug infusion, reported that MDMA caused long-lasting suppression of both dopamine and 5-HT neurons, through its activity on dopamine and 5-HT autoreceptors [68]. 

In our experimental conditions, MDMA and NT did not co-stain with microglia and astrocytes, at least within 24 h after MDMA administration. However, the important pathological link existing among MDMA administration, activation of neuroinflammatory pathways in the CNS [69], and glia activation [8] must be taken adequately into account. Indeed, it has been reported that administration of neurotoxic amphetamines significantly increased both resting and activated microglia, as well as astrocytes, causing enhanced production of reactive species, such as NO [70,71], and increased release of proinflammatory cytokines [72,73]. Thus, we cannot exclude the possibility that the involvement of the inflammation-associated cellular subtypes of the CNS (i.e., microglia and astrocytes) might occur later with respect to our chosen time point (24 h from MDMA administration).

Here, by analyzing the effects of MDMA at three specific time points, namely, 6 h, 16 h, and 24 h after its administration, we also defined a possible time course for the onset of its neurotoxic effects. This is an innovative experimental approach designed by our research group, allowing to evaluate if MDMA-induced alterations in the CNS might occur within the first 24 h from its administration Importantly, we did not detect significant differences in the analyzed parameters within the group of MDMA-exposed animals with respect to the different considered time points, as we already observed an increased expression of iNOS and NT staining after 6 h from the injection of this psychoactive compound, suggesting an early onset of the dysfunctions induced by MDMA-derived oxidative stress, without differences with respect to later time points. Future investigations at earlier time points (i.e., after 1 or 2 h from MDMA administration) might be useful to further define the induction profile of the enzymatic systems implicated in MDMA-induced neurotoxicity.

An important aspect of our study is that all findings were observed in the absence of body temperature elevations. Indeed, in our experimental protocol, MDMA was administered at 18 °C, this temperature preventing the hyperthermic response [74] described, instead, at standard housing temperature (21–23 °C) [57,75,76]. Therefore, the observed effects of MDMA administration on the expression of oxidative and nitrosative stress producing enzymes might be considered, at least in our experimental conditions, independently from MDMA-induced hyperthermia. Although it has been reported that hyperthermia is a significant complication of MDMA use and a factor potentiating its toxic effects on the CNS [77], several other mechanisms underlying MDMA-induced neurotoxicity have been described, such as altered oxidase metabolism of monoamines, glutamate excitotoxicity, agonism of 5-HT 2A receptors, as well as formation of neurotoxic metabolites [52].

The most important limitation of this work is related to the lack of a direct measure of iNOS activation and RNS amount, produced following MDMA administration. However, we found that the increased iNOS immunoreactivity, detected in MDMA-exposed animals, was occurring along with elevations in the number of NT-positive stained cells. Elevations in this marker may suggest a possible increase in the activation and functioning of the iNOS enzyme. Indeed, NT is known to be a direct marker of nitrosative stress, being the product of tyrosine nitration mediated by RNS such as peroxynitrite anion and nitrogen dioxide, formed in the presence of NO [78]. In the same line, further investigations, performed by using specific techniques or other tissue fixation conditions [79,80,81,82,83], are needed to directly evaluate ROS amount in the brain following NOX enzyme activation.

Together with the induction of neuropathological alterations in CNS, psychoactive compounds are known to induce significant behavioral changes [84,85,86]. Thus, another important limitation of this study regards the lack of behavioral analyses on MDMA-exposed rats. Previously preclinical published papers reported that MDMA administration was able to alter sexual behavior [87], to induce anxiety-like and avoidant behaviors [88], to reduce social interactions, also enhancing the rewarding effect of other drugs of abuse [89] in rodents. Thus, despite the absence of behavioral data in the present work, the important pathogenetic role that iNOS has been reported to play in the modulation of these behaviors [51,90,91,92] may represent a link between our immunohistochemical findings and the previously described MDMA-induced behavioral impairment.

This study may have important clinical implications. Indeed, MDMA has been described as one of the three most commonly used illicit substances [93] and still remains a very popular psychostimulant drug, especially among young adults, aged 18–25 years (https://www.drugabuse.gov/drugs-abuse/mdma-ecstasymolly), (https://www.theguardian.com/uk-news/2015/jul/23/ecstasy-and-lsd-use-reaches-new-high-among-young) [94]. The search of novel biomarkers and/or pharmacological strategies to reduce the neurological burden associated with the misuse of this compound represents, therefore, a clinical priority. Thus, increased iNOS expression and nitrosative stress described in our study may be considered as novel targets to be used for the clinical monitoring and the pharmacological treatment of MDMA’s detrimental effects on the CNS. Moreover, as contrasting results have been published about the possible relationship between MDMA and neurocognitive deficits [95], the identification of novel molecular pathways underlying the impact of this substance abuse may provide novel insights into this crucial issue. 

## 4. Materials and Methods

### 4.1. Animals

Adult (8–10 weeks), male Wistar rats (Envigo, San Pietro al Natisone, Italy) weighing 200–250 g were housed at constant room temperature (22 ± 1 °C) and relative humidity (55 ± 5%) under a 12 h light/dark cycle (lights on from 7:00 AM to 7:00 PM) for at least seven days before the experiments. Food and water were available ad libitum. Procedures involving animals and their care were conducted in strict accordance with the European (86/609/EEC) and Italian (DLgs 116/92; notice pursuant to art. 7) guidelines on animal care. The Guide for the Care and Use of Mammals in Neuroscience and Behavioral Research (National Research Council 2004) was also followed. All experimental techniques and scientific procedures involving animals were conducted in accordance with “Animal Research: Reporting of In Vivo Experiments” (ARRIVE) guidelines. All efforts were made to minimize the number of animals used and to alleviate their suffering. Animal welfare was daily monitored through the entire period of experimental procedures.

### 4.2. MDMA Administration and Experimental Protocol

The MDMA dose (20 mg/kg) and way of administration (intraperitoneally, i.p.) used were chosen based on previously published works from our group, as well as others [36,37,96].

In order to avoid the MDMA-induced hyperthermic response [74] and in order to exclude the possibility that the observed effects might be only related to body temperature elevation, animals were conducted from the standard housing room (21–23 °C), where they stayed for at least seven days before the experiments, to the 18 °C room one hour before MDMA administration, in order to acclimatize to the lower temperature. We monitored animal temperature before and 30 minutes after MDMA administration [57] by using a rectal probe and we did not observe any hyperthermic response in any of the MDMA-exposed animals. A total of 24 animals were initially exposed to MDMA and a total of 9 animals to saline (vehicle). The total number of animals to be included in this study was established based on our previous experience with immunohistochemical analyses on rodent brain [27,97,98,99] and on previous immunohistochemical studies on rat brain from other groups [100,101,102]. Four animals died within the first hour from MDMA administration. The 29 remaining animals were killed by decapitation after 6 h (MDMA *n* = 8; saline = 3), 16 h (MDMA *n* = 7; saline = 3), or 24 h (MDMA *n* = 5; saline = 3) from MDMA or saline administration. No significant differences were detected in the considered parameters within saline-exposed animals (data not shown). Therefore, results regarding saline-exposed animals are presented as one single control (CTRL) group.

### 4.3. Immunohistochemical Analyses

After rat decapitation, the entire brain was removed and fixed in 10% buffered formalin for 48 h [39]. The fixed brain was dissected in four specimens of about 20 mm (named as A, B, C, and D), proceeding respectively from the anterior to the posterior regions of the rat brain. Each of these sections was then processed for paraffin inclusion. For this study, 4 μm paraffin-embedded sections of the frontal cortex region were obtained from specimen A, by using an automized microtome (Leica, Cambridge, UK), mounted on 3-amminopropyl-triethoxysilane covered slides (Fluka, Buchs, Switzerland), and dried at 37 °C for 24 h. Brain sections were then deparaffinized through graded alcohols, subjected to epitope retrieval for 15 min and incubated for two hours at room temperature with primary antibodies, diluted in a blocking buffered serum solution containing albumin and fetal bovine serum (Sigma-Aldrich S.R.L., Milan, Italy), raised against NOX2 (1:50, Santa Cruz Biotechnology, Inc., Dallas, TX, USA), NOX1 (1: 250, Abcam, Cambridge, UK), NOX4 (1:100, Abcam), iNOS (1:100, Santa Cruz Biotechnology), eNOS, (1:100, Santa Cruz Biotechnology), nNOS (1:150, Santa Cruz Biotechnology, Inc.), 8OHdG (1:10, JaICA, Shizuoka, Japan), NT (1:600, Santa Cruz Biotechnology, Inc.), DT1 (1:100, Abcam). Sections were then washed with phosphate-buffered saline (PBS) and incubated for 15 min at room temperature with specific biotinylated secondary antibodies. After several washes in PBS, sections were incubated for 15 min in horseradish peroxidase-avidin/biotin complex solution. Horseradish peroxidase was visualized using 3,3-diaminobenzidinetetrahydrochloride hydrate (DAB, Sigma-Aldrich S.R.L.) and H_2_O_2_. Counterstaining with hematoxylin-eosin allowed visualization of cell morphology and nuclei by light microscopy. Specificity of NOX2 and iNOS antibodies was previously investigated by our research group on positive and technical negative controls [103].

The MDMA immunostaining was performed as previously described [39], by using a monoclonal antibody that specifically recognizes MDMA (clone 1A9, kindly supplied by Microgenics GmbH Products Europe, Passau, Germany). Specificity of this primary antibody was tested on positive and negative controls (brain samples of MDMA- and saline-exposed rats, respectively), as well as technical negative controls (without primary antibody), by using different experimental conditions, that is, both without pretreatment and with several pretreatments (boiling in 0.25 mM EDTA buffer; boiling in 0.1 M citric acid buffer; proteolytic enzyme at 20 °C for 5 min; proteinase K at 20°C for 15 min), at various concentrations (ratio 1:20, 1:50, 1:100, 1:500, 1:1000, 1:2000). The tested samples were examined under a light microscope in order to detect the best reaction, that is, anti-MDMA concentration 1:100, with a boiling pre-treatment in 0.25 mM EDTA buffer. The detection system used for the MDMA immunostaining was the LSAB1 Kit (Dako, Carpinteria, CA, USA).

Double immunohistochemistry for MDMA/DT1, NT/DT1, and MDMA/NOX2 was performed as previously described [104]. Briefly, slices were incubated with the above-cited primary antibodies. After the incubation, the peroxidase–avidin/biotin complex was visualized using the following peroxidase substrates with different colors: Vector NovaRED (red, Vector, Burlingame, CA, USA), Vector VIP (purple, Vector, Burlingame, CA, USA), and Vector SG (blue/grey, Vector, Burlingame, CA, USA). Sections were counterstained with methyl green, dehydrated, coverslipped, and observed in a Leica DM6000 optical microscope (Leica, Cambridge, UK). The different combinations of colors used for each above-mentioned double immunohistochemistry are detailed in Table 1.

Double immunofluorescence for DT1/NeuN (DT1 = 1:100; NeuN = 1:1000, Abcam, Cambridge, UK), MDMA/IBA-1 (MDMA = 1:100; IBA-1 = 1:500, kindly provided by Prof. Livio Luongo, Department of Experimental Medicine Division of Pharmacology University of Campania “L. Vanvitelli” Naples, Italy), MDMA/GFAP (MDMA = 1:100; GFAP = 1:500, kindly provided by Prof. Livio Luongo, Department of Experimental Medicine Division of Pharmacology University of Campania “L. Vanvitelli” Naples, Italy), NT/IBA-1, and NT/GFAP was performed as previously described [97], using the following fluorescent secondary antibodies: goat anti-rabbit ALEXA Fluor Plus 555 (1:1000, ThermoFisher Scientific, Milan, Italy) for DT1, IBA-1, and GFAP staining, goat anti-mouse ALEXA Fluor Plus 488 (1:1000, ThermoFisher Scientific) for NeuN, MDMA, and NT staining. Sections were counterstained with 4′, 6-diamidino-2-phenylindole (DAPI, ThermoFisher Scientific) in order to stain cellular nuclei, coverslipped, and observed using a Nikon Ti-E time-lapse microscope (Nikon, Campi Bisenzio, Italy).

Quantification of NOX2, NOX1, NOX4, iNOS, eNOS, nNOS, 8OHdG, and NT positive-stained cells was performed by the ImageJ software (imagej.nih.gov/ij/), as previously described [103], using the “Manual Cell Counting and Marking” protocol of this software for RGB color, single, not stack images (https://imagej.nih.gov/ij/docs/guide/user-guide.pdf). One image for each animal of the different experimental groups was processed. Quantifications were expressed as number of positive-stained cells/analyzed area. 

### 4.4. Blindness of the Study

Histological analyses were performed by researchers who were blind with respect to the treatment conditions. The blinding of the data was maintained until the analysis was terminated.

### 4.5. Statistical Analysis

Data were analyzed using the GraphPad Prism 5 software for Windows (La Jolla, CA, USA). Data were checked for normality using Bartlett’s test and analyzed by one-way analysis of variance (ANOVA), followed by Tukey’s post-hoc test. For all tests, a *p* value <0.05 was considered statistically significant. Results are expressed as means ± mean standard error (SEM).

## 5. Conclusions

In conclusion, our study suggests a crucial and specific role of nitrosative stress in the development of neuropathological alterations induced by ecstasy consumption, and may represent significant progress in the understanding of its neurodetrimental effects. Moreover, the identification of iNOS as the most involved isoform of the NO synthase enzyme may open novel insights into the identification of specific biomarkers of the early phases of MDMA-induced neurotoxicity, thus adding an important piece of information about the neuroactivity of this drug and into the development of effective targeted pharmacological approaches, inhibiting the production of nitrogen reactive species. 

## Figures and Tables

**Table 1:** Combinations of colors used in double immunohistochemistry experiments.

Antibodies	Colors
**MDMA/DT1**	Red/Blue-grey
**NT/DT1**	Purple/Blue-grey
**MDMA/NOX2**	Purple/Blue-grey

## Supplementary Materials

Supplementary materials can be found at https://www.mdpi.com/1422-0067/20/5/1242/s1.
