# Supplementary material for: Increased iNOS and Nitrosative Stress in Dopaminergic Neurons of MDMA-Exposed Rats
Source: Int J Mol Sci. 2019 Mar 12;20(5):1242. doi: 10.3390/ijms20051242 (PMC6429174; doi:10.3390/ijms20051242)
Supplement: Supplementary file 1 [file ijms-20-01242-s001.pdf]

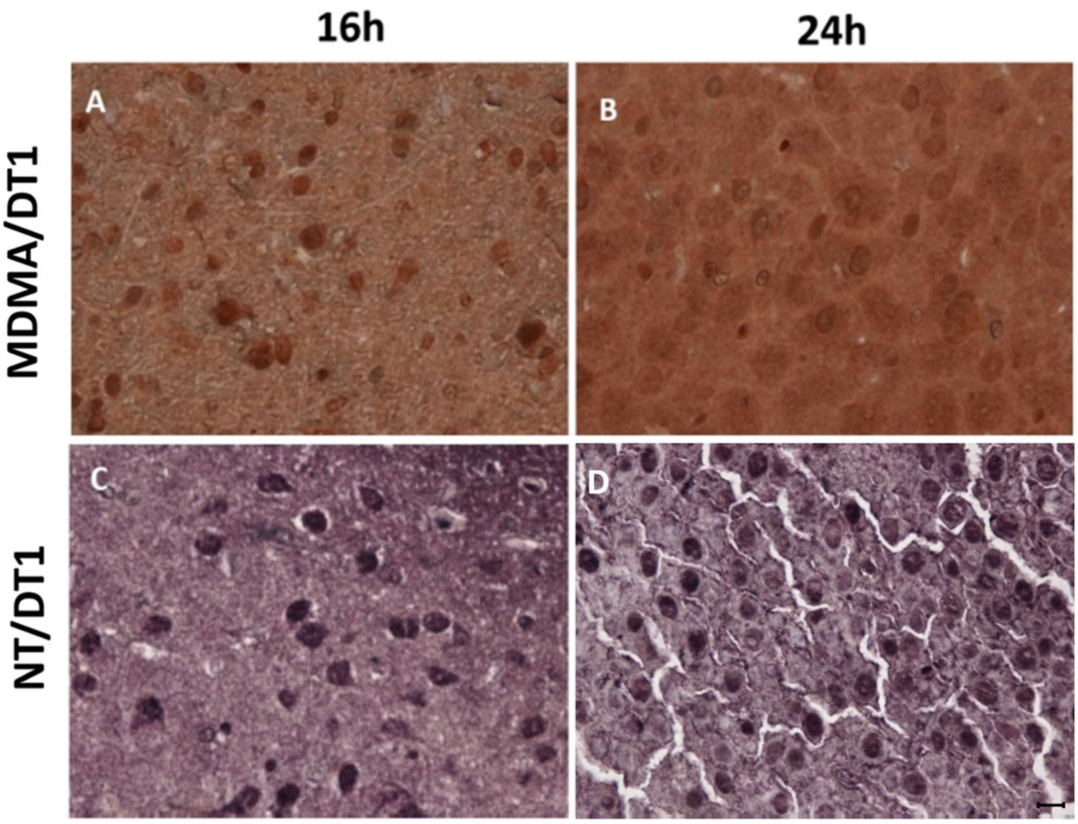

Figure S1. MDMA/DT1 and NT/DT1 double immunostaining in rats receiving MDMA and sacrificed after 16 or 24 h. (**A**,**B**) Representative images (light microscopy, 40×) of MDMA/DT1 double immunostaining in the frontal cortex of rats receiving MDMA and sacrificed after (**A**) 16 h and (**B**) 24 h from its administration. (**C**,**D**) Representative images (light microscopy, 40×) of NT/DT1 double immunostaining in the frontal cortex of rats receiving MDMA and sacrificed after (**C**) 16 h and (**D**) 24 h from its administration. Scale bar for images in panels (**A**–**D**) = 50 μm.

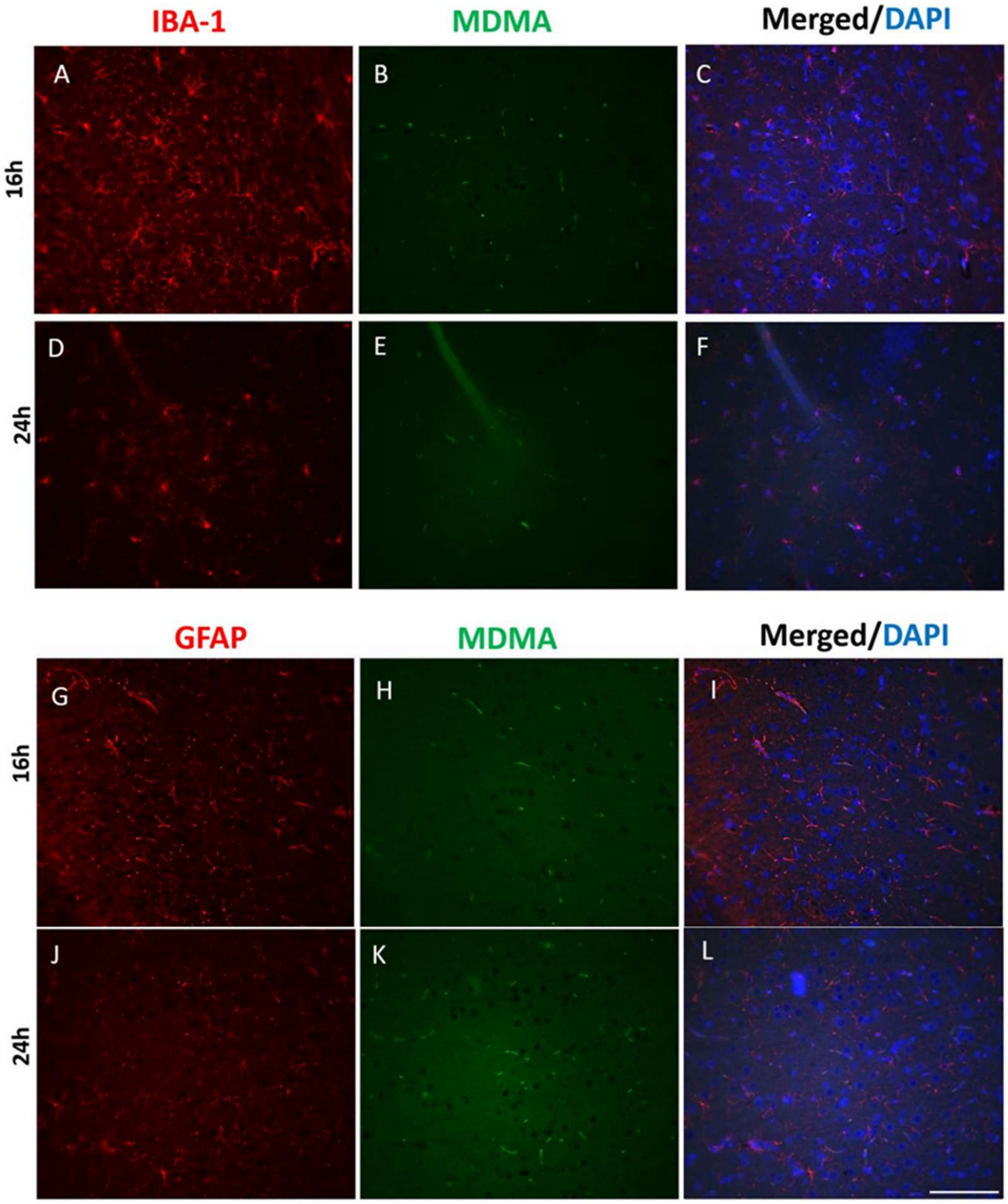

Figure S2. IBA-1/MDMA and GFAP/MDMA double immunofluorescence in rats receiving MDMA and sacrificed after 16 or 24 h. (**A**–**C**) Representative immunofluorescence images (light microscopy, 40×) of IBA-1 (**A**-red staining)/MDMA (**B**-green staining) and merged images with DAPI (**C**-blue staining) in the frontal cortex of rats receiving MDMA and sacrificed after 16 h from its administration. (**D**–**F**) Representative immunofluorescence images (light microscopy, 40×) of IBA-1 (**D**-red staining)/MDMA (**E**-green staining) and merged images with DAPI (**F**-blue staining) in the frontal cortex of rats receiving MDMA and sacrificed after 24 h from its administration. (**G**–**I**) Representative immunofluorescence images (light microscopy, 40×) of GFAP (**G**-red staining)/MDMA (**H**-green staining) and merged images with DAPI (**I**-blue staining) in the frontal cortex of rats receiving MDMA and sacrificed after 16 h from its administration. (**J**–**L**) Representative immunofluorescence images (light microscopy, 40×) of GFAP (**J**-red staining)/MDMA (**K**-green staining) and merged images with DAPI (**L**-blue staining) in the frontal cortex of rats receiving MDMA and sacrificed after 24 h from its administration. Scale bar for images in panels (**A**–**L**) = 100 μm.

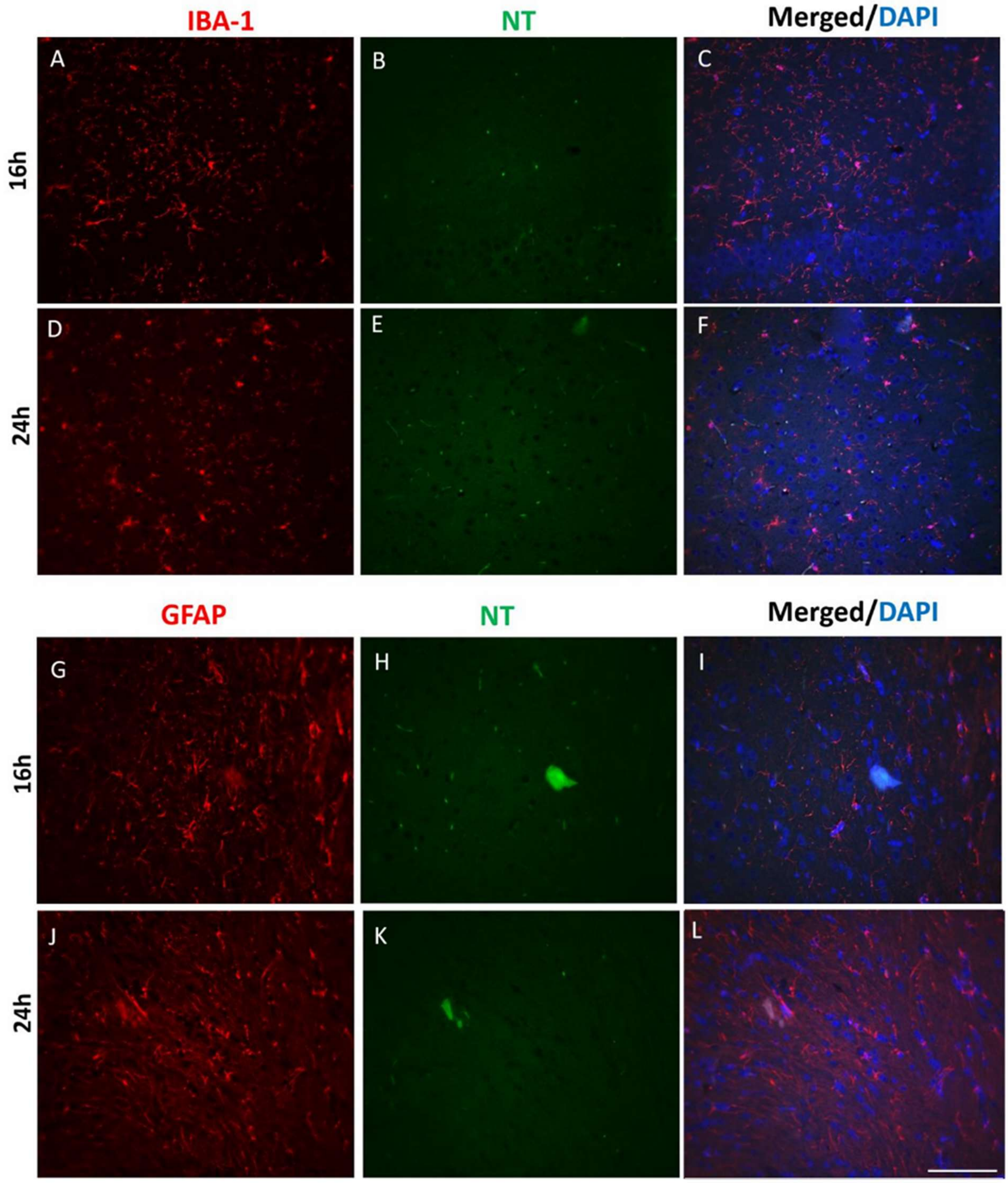

Figure S3. IBA-1/NT and GFAP/NT double immunofluorescence in rats receiving MDMA and sacrificed after 16 or 24 h. (**A**–**C**) Representative immunofluorescence images (light microscopy, 40×) of IBA-1 (**A**-red staining)/NT (**B**-green staining) and merged images with DAPI (**C**-blue staining) in the frontal cortex of rats receiving MDMA and sacrificed after 16 h from its administration. (**D**–**F**) Representative immunofluorescence images (light microscopy, 40×) of IBA-1 (**D**-red staining)/NT (**E**-green staining) and merged images with DAPI (**F**-blue staining) in the frontal cortex of rats receiving MDMA and sacrificed after 24 h from its administration. (**G**–**I**) Representative immunofluorescence images (light microscopy, 40×) of GFAP (**G**-red staining)/NT (**H**-green staining) and merged images with DAPI (**I**-blue staining) in the frontal cortex of rats receiving MDMA and sacrificed after 16 h from its administration. (**J**–**L**) Representative immunofluorescence images (light microscopy, 40×) of GFAP (**J**-red staining)/NT (**K**-green staining) and merged images with DAPI (**L**-blue staining) in the frontal cortex of rats receiving MDMA and sacrificed after 24 h from its administration. Scale bar for images in panels (**A**–**L**) = 100 µm.

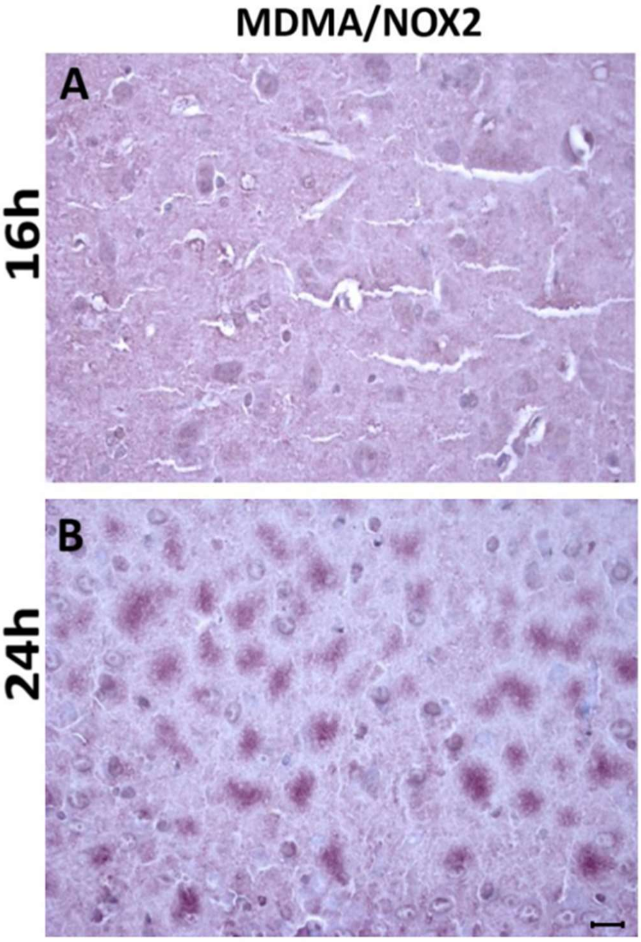

Figure S4. MDMA/NOX2 double immunostaining in rats receiving MDMA and sacrificed after 16 or 24 h. (**A**,**B**) Representative images (light microscopy, 40×) of MDMA/NOX2 double immunostaining in the frontal cortex of rats receiving MDMA and sacrificed after (**A**) 16 h and (**B**) 24 h from its administration. Scale bar for images in panels (**A**,**B**) = 50 μm.
